# Supplementary material for: Stenting in Brain Hemodynamic Injury of Carotid Origin Caused by Type A Aortic Dissection: Local Experience and Systematic Literature Review
Source: J Pers Med. 2022 Dec 27;13(1):58. doi: 10.3390/jpm13010058 (PMC9861720; doi:10.3390/jpm13010058)
Supplement: Supplementary file 1 [file jpm-13-00058-s001.zip › jpm-2121126-supplementary.pdf]

SUPPLEMENTAL MATERIAL :

- Supplemental Table S1: Risk of bias of each case report study according to the The Joanna Briggs Institute Critical Appraisal tools.

- Supplemental Table S2: Risk of bias of each individual non-randomised study according to the Cochrane Collaboration tool.

- Supplemental Table S3A : Individual patients data from the patients treated by aortic and carotid surgery

- Supplemental Table S3B : Individual patients data from the patients treated by carotid stenting before aortic surgery

- Supplemental Table S3C : Individual patients data from the patients treated by carotid stenting after aortic surgery

Supplemental Table S1: Risk of bias of each case report study according to the The Joanna Briggs Institute Critical Appraisal tools.									
**Study / Criterion**	1	2	3	4	5	6	7	8	Overall
Published as full papers									
**Schönholz et al. 2008**	Unclear	No	Yes	Unclear	Yes	Yes	No	Yes	Include
**Chahine et al. 2018**	Yes	Yes	Yes	Yes	Unclear	Unclear	No	Unclear	Include
**Morihara et al. 2016**	Unclear	Unclear	Yes	Yes	Yes	Yes	No	Yes	Include
**Matsumoto et al. 2016**	Unclear	Yes	Yes	Yes	Unclear	Yes	Yes	Yes	Include
**Amr et al. 2016**	Yes	Yes	Yes	Yes	Yes	Yes	yes	Yes	Include
**Hong et al. 2005**	Unclear	Unclear	Yes	Yes	Yes	Yes	Yes	Yes	Include
**Karawabuki et al. 2006**	Unclear	Yes	Yes	Yes	Yes	Unclear	Yes	Yes	Include
**Kim et al. 2006**	Unclear	Yes	Yes	Yes	Yes	Yes	Yes	Yes	Include
**Ueyama et al. 2007**	Unclear	No	Unclear	Yes	Yes	Yes	No	Yes	Include
**Roseborough et al. 2006**	Yes	No	Yes	Yes	Yes	Yes	Yes	Yes	Include
**Sakaguchi et al. 2005**	Unclear	No	Yes	Yes	Yes	Unclear	No	Yes	Include
**Usui et al. 2021**	Yes	Yes	Yes	Yes	Unclear	Yes	No	Yes	Include
**Fukuhara et al. 2021**	Unclear	No	Yes	Yes	Unclear	Yes	No	Yes	Include
**Funakoshi et al. 2020**	Unclear	Yes	Yes	Yes	Yes	Yes	Yes	Yes	Include
**Heran et al. 2019**	Unclear	Unclear	Yes	Yes	Yes	Yes	No	Yes	Include
**Popovic et al. 2016**	Yes	Yes	Yes	Yes	Yes	Yes	Yes	Yes	Include
**Casana et al. 2011**	Yes	Yes	Yes	Yes	Yes	Yes	Yes	Yes	Include

Supplemental Table S2: Risk of bias of each individual non-randomised study according to the Cochrane Collaboration tool.									
**Study / Criterion**	1	2	3	4	5	6	7	8	Overall
Published as full papers									
**Fichadaya et al. 2022**	/	+	+	/	+	+	++	/	Mild
**Morimoto et al. 2011**	++	+	+	++	+	+	+	+	Low

Criteria used

1. Was selection of exposed and non-exposed cohorts drawn from the same population?
2. Can we be confident in the assessment of exposure?
3. Can we be confident that the outcome of interest was not present at start of study
4. Did the study match exposed and unexposed for all variables that are associated with the outcome of interest or did the statistical analysis adjust for these prognostic variables?"
5. Can we be confident in the assessment of the presence or absence of prognostic factors?
6. Can we be confident in the assessment of outcome?
7. Was the follow up of cohorts adequate?
8. Were co-Interventions similar between groups?

Ratings

Definitely yes (Low risk of bias) ++
Probably Yes +
Probably No -
Definitely No (High risk of Bias) --

Supplemental Table S3A : Individual patients data from the patients treated by aortic and carotid surgery

Article	Schönholz et al.	Chahine et al.	Morihara et al.	Matsumoto et al. Patient 1	Matsumoto et al. Patient 2	Amr et al. Patient 1	Amr et al. Patient 2	Hong et al.	Kawarabuki et al.	Kim et al.	Ueyama et al.	Sakaguchi et al.	Usui et al.	Fukuhara et al.	Fichadiya et al. **10 patients**	Morimoto et al. **41 patients**
Demographics																
Age (y) mean(sd)	52	44	60	63	73	79	65	69	64	30	72	56	65	77	60.5(12)	68(10)
Sex : Male(M) Female (F)	M	F	M	F	M	F	F	F	F	M	F	M	F	M	NA	NA
Arterial hypertension	NA	Yes	NA	NA	NA	Yes	Yes	NA	Yes	Yes	NA	NA	No	NA	NA	36(87%)
Current smoking	NA	Yes	NA	NA	NA	NA	NA	NA	NA	NA	NA	NA	NA	NA	NA	NA
Pre-stroke mRS	1	1	1	1	1	1	1	1	1	1	1	1	1	1	NA	NA
Clinical																
NIHSS	NA	0	16	16	20	NA (syncope, left hemiplegia and aphasia)	NA (left hemiplegia)	6	11	18	NA	NA (Glasgow 14)	2	37	NA	10.7(8)
Management																
Time from symptom onset to surgery ; mean (SD)	NA	NA	Day5	NA	NA	25h	2h	4h	7h	3h	7h	2h	2.5h	12h	NA	21,7h ; (40,5h)
Radiological																
ASPECTS Score	NA	7	NA(watershed infarcts)	9	1	10	10	10	10	10	10	10	10	10	NA	10
Dissected arteries	RCCA	RCCA and ICA, LCCA and ICA	RCCA and ICA	RCCA and ICA	RCCA	RCCA	RCCA	RCCA, LCCA	RCCA and ICA	RCCA and ICA, LCCA	RCCA and ICA	RCCA	RCCA and ICA	RCCA, LCCA	40% RCCA 40% LCCA	NA
Aftermath																
Survival Yes/No	No(death during surgery)	Yes	Yes	Yes	No (brain herniation post surgery)	Yes	Yes	Yes	Yes	Yes	Yes	Yes	Yes	Yes	21% death	6(14%) death
Deficit regression post ACS	/	NA	Complete	Complete	/	Complete	Complete	Complete	Complete	Complete	Complete	Complete	Partial	Partial	NA	26(63%)
New neurological deficit	/	No	No	No	/	No	No	No	No	No	No	No	No	No	NA	NA

New lesion on imaging	/	No	No	NA	/	No	Yes	Yes	NA	Yes	No	NA	NA	NA	NA	23(56%)
mRS score after 24h	/	0	NA	NA	/	NA	NA	NA	0	0	NA	NA	NA	NA	NA	NA
mRS score at discharge	/	0	NA	0	/	0	0	0	0	0	0	0	0	1	NA	NA
mRS score on follow-up	/	1	NA	NA	/	0	0	0	NA	NA	NA	NA	NA	NA	NA	NA

Supplemental Table S3B : Individual patients data from the patients treated by carotid stenting before aortic surgery

Article/Patient	Local center patient 1	Local center patient 2	Local center patient 3	Local center patient 4	Funakoshi et al. Patient 1	Funakoshi et al. Patient 2	Heran et al.	Popovic et al.
Demographics								
Age (y)	77	71	72	72	63	57	73	37
Sex : Male(M) Female (F)	M	M	M	M	F	F	M	M
Arterial hypertension	Yes	No	Yes	Yes	NA	No	NA	Yes
Current smoking	No	No	Yes	No	NA	NA	NA	Yes
Pre-stroke mRS	0	0	0	0	0	0	0	0
Clinical								
NIHSS	21	NA(left hemiplegia)	10	3	24	18	NA(left hemiparesis, Hemilatral anopsia, hemi- neglect)	10
Management								
Time from symptom onset to angioplasty	2h	6h	Day2(initial monitoring only)	18h	NA	NA	5h	3h
Radiological								
ASPECTS Score	7	10	8	9	10	8	10	10
Dissected arteries	RCCA and ICA, LCCA and ICA	RCCA and ICA	RCCA and ICA	RCCA	RCCA, LCCA, R vertebral artery	RCCA	RCCA	RCCA
Per-procedure complications (CS)	No	No	No	No	No	No	No	No
Aftermath								
Deficit regression post CS	Partial	Partial	Partial	No	Partial	Partial (almost complete regression)	Partial	NA
New lesion on post CS imaging	No	NA	No	Yes (single ischemic spot)	Yes (small signal hyperintensity areas in the left cerebral cortex)	No	NA	NA
Delay CS-Surgery	2h	NA(death day2 mesenteric ischemia)	NA(previous Bentall surgery)	Same day	Same day	12days	Immediate	8h
Survival Yes/No	Yes	No	Yes	Yes	Yes	Yes	Yes	No (death from mediastinits at day35)
New neurological deficit	No	/	No	No	No	No	No	NA
New lesion on post surgery imaging	No	/	NA	No	NA	NA	NA	NA
mRS score at discharge	2	/	4	NA	NA	NA	1	/
mRS score on follow-up	1	/	3	1	1	1	1	/
Stent permeability on follow-up	Yes	/	Yes	Yes	NA	NA	Yes	/

Supplemental Table S3C : Individual patients data from the patients treated by carotid stenting after aortic surgery

Article/Patient	Local center patient 5	Local center patient 6	Local center patient 7	Local center patient 8	Local center patient 9	Roseborough et al.	Casana R et al.
Demographics							
Age (y)	78	64	71	74	62	66	66
Sex : Male(M) Female (F)	F	M	F	F	F	M	F
Arterial hypertension	Yes	Yes	Yes	No	Yes	NA	No
Current smoking	No	Yes	No	No	Yes	NA	NA
Pre-stroke mRS	0	0	0	0	0	0	0
Clinical							
NIHSS	NA(left hemiplegia, Glasgow 8)	0	0	0	0	0	0
Management							
Time from symptom onset to surgery	6h	6h	6h	4h	8h	2days	NA
Radiological							
ASPECTS Score (initial)	10	10	NA	NA	NA	NA	10
Dissected arteries	RCCA	LCCA	RCCA	RCCA and ICA, LCCA	RCCA	LCCA and ICA	RCCA and ICA, LCCA
Surgery aftermath							
New neurological deficit	Yes (anisocoria and left hemiplegia)	Yes (ataxia and left arm paresis)	Yes (Tonic-clonic seizure)	Yes(confusion)	Yes (left hemiplegia and hemineglegence)	Yes (Absence of movements of left side)	Yes (drop attacks)
New lesion on post-op imaging	Yes (complete right anterior and sylvian stroke)	Yes (right junctionnal ischemic lesions and hemispheric hypoperfusion)	Yes (bilateral watershed infarcts, right hemisphere hypoperfusion)	No (isolated right hemisphere hypoperfusion)	Yes (right junctionnal ischemic lesions and hemisphere hypoperfusion)	Yes (right small watershed infarcts, right hemisphere hypoperfusion)	No
Delay Surgery-CS	1day	1day	4h	14days	3days	4days	30days
Stenting aftermath							
Per-procedure complications (CS)	Yes(common carotid intra-stent thrombosis treated by immediate aspiration, A2 distal emboli treated by immediate aspiration)	No	No	Yes (right sylvian M2 embolism, treated by thrombectomy)	Yes (distal emboli)	No	No
Deficit regression post CS	No	Partial	Partial	No	No	Partial	Partial
New neurological deficit	No	Yes (transient majoration due to reperfusion oedema)	No	No	No	No	No
New lesion on post CS imaging	No	No	NA	Yes (small right waterhed infarcts)	Yes (diffuse ischemic lesions)	No	NA
Survival Yes/No	No (Neurologic degradation day8)	Yes	Yes	No (cardio-respiratory arrest day4)	Too early	Yes	Yes
mRS score at discharge	NA	1	4	NA	Too early	2	NA
mRS score on follow-up	NA	1	1	NA	Too early	0	NA

Stent permeability on follow-up	Yes	Yes	Yes	Yes	Too early	Yes	Yes
